# Cordycepin Inhibits Virus Replication in Dengue Virus-Infected Vero Cells

**DOI:** 10.3390/molecules26113118

**Published:** 2021-05-23

**Authors:** Aussara Panya, Pucharee Songprakhon, Suthida Panwong, Kanyaluck Jantakee, Thida Kaewkod, Yingmanee Tragoolpua, Nunghathai Sawasdee, Vannajan Sanghiran Lee, Piyarat Nimmanpipug, Pa-thai Yenchitsomanus

**Affiliations:** 1 Center of Excellence for Innovation in Analytical Science and Technology, Chiang Mai University, Chiang Mai 50200, Thailand; aussara.pan@cmu.ac.th; 2 Department of Biology, Faculty of Science, Chiang Mai University, Chiang Mai 50200, Thailand; mildmsp@gmail.com (S.P.); kanyaluckjan@gmail.com (K.J.); tda007suju@gmail.com (T.K.); yboony150@gmail.com (Y.T.); 3 Division of Molecular Medicine, Research Department, Faculty of Medicine Siriraj Hospital, Mahidol University, Bangkok 10700, Thailand; tay_pcr@hotmail.com (P.S.); sawasdee111@gmail.com (N.S.); 4 Department of Chemistry, Faculty of Science, University of Malaya, Kuala Lumpur 50603, Malaysia; vannajan@um.edu.my; 5 Department of Chemistry, Faculty of Science, Chiang Mai University, Chiang Mai 50200, Thailand

**Keywords:** bioactive compound, cordycepin, cordyceps extract, dengue virus, antiviral activity

## Abstract

Dengue virus (DENV) infection causes mild to severe illness in humans that can lead to fatality in severe cases. Currently, no specific drug is available for the treatment of DENV infection. Thus, the development of an anti-DENV drug is urgently required. Cordycepin (3′-deoxyadenosine), which is a major bioactive compound in Cordyceps (ascomycete) fungus that has been used for centuries in Chinese traditional medicine, was reported to exhibit antiviral activity. However, the anti-DENV activity of cordycepin is unknown. We hypothesized that cordycepin exerts anti-DENV activity and that, as an adenosine derivative, it inhibits DENV replication. To test this hypothesis, we investigated the anti-DENV activity of cordycepin in DENV-infected Vero cells. Cordycepin treatment significantly decreased DENV protein at a half-maximal effective concentration (EC50) of 26.94 μM. Moreover, DENV RNA was dramatically decreased in cordycepin-treated Vero cells, indicating its effectiveness in inhibiting viral RNA replication. Via in silico molecular docking, the binding of cordycepin to DENV non-structural protein 5 (NS5), which is an important enzyme for RNA synthesis, at both the methyltransferase (MTase) and RNA-dependent RNA polymerase (RdRp) domains, was predicted. The results of this study demonstrate that cordycepin is able to inhibit DENV replication, which portends its potential as an anti-dengue therapy.

## 1. Introduction

Dengue virus (DENV) infection is one of the most rapidly spreading arboviral diseases of humans in the world [1]. It is a major health problem among those dwelling in tropical and subtropical areas, and it is spreading globally, with approximately 129 countries now thought to be DENV-endemic areas [2]. The incidence of DENV infection continues to increase. An estimated 100–400 million people are infected annually, and 96 million people require medical care [3]. There is currently no specific drug to treat DENV infection. Supportive treatment is the only treatment option for hospitalized DENV-infected patients.

DENV is a member of the Flaviviridae family, which includes four closely related serotypes, including DENV1, DENV2, DENV3, and DENV4 [1]. All four serotypes are usually cocirculating in DENV-endemic areas, and the virus infects humans via the bite of female *Aedes aegypti* or *Aedes albopictus* mosquitoes, causing clinical manifestations that vary from mild to severe [4]. Subsequent infections with serotypes different from the primary infection were reported to promote severe disease, and this is the major obstacle to DENV vaccine development [4]. A tetravalent vaccine against all four DENV serotypes represents the ideal model for protecting against DENV infection and minimizing its severity [5]. Attempts to develop an effective dengue vaccine have been ongoing for decades, and these attempts have yielded one currently available licensed vaccine. Sanofi Pasteur’s Dengvaxia^®^ vaccine has been distributed to vaccinate people against DENV infection in more than 20 countries worldwide [6]. However, the overall efficacy of this vaccine was reported to be limited with suboptimal protection against DENV1 and DENV2 (50% and 35–42% protection, respectively) [7,8,9]. Although the availability of a DENV vaccine is necessary for the prevention and control of viral infection, anti-DENV drugs for the treatment of DENV infection are also important for preventing disease progression, for reducing disease severity, and for interrupting the spread of the virus.

Biological resources in and from nature harbor large and diverse assortments of bioactive compounds [10]. Natural compounds from herbs and medicinal plants are recognized for their safety and effectiveness, and many of them have been used as traditional medicines by people from many countries around the world. Since these natural compounds often contain a broad spectrum of biological activities, they have the potential to be further purified and developed into drugs for the treatment of many human disease conditions. Cordyceps (ascomycete) fungus has been used in Chinese medicine since ancient times [11]. The pharmacological properties of Cordyceps extract have been studied in both infectious and noninfectious diseases [12]. Cordycepin (3′-deoxyadenosine) is an adenosine derivative, and a major bioactive compound in *Cordyceps sinensis* and *Cordyceps militaris*. This compound was previously reported to have antiviral activity [13,14,15,16,17]. However, the anti-DENV activity of cordycepin is unknown. Since cordycepin is an adenosine derivative, we hypothesized that cordycepin has anti-DENV activity and is likely to inhibit DENV replication. The results of this study clearly demonstrate that cordycepin inhibits DENV replication in African green monkey kidney (Vero) cells and also decreases DENV RNA synthesis. This inhibitory effect was also observed when a *C. militaris* extract was similarly tested.

## 2. Results

### 2.1. Cordycepin Inhibited DENV2 Infection after Cellular Entry

**Figure 1:**
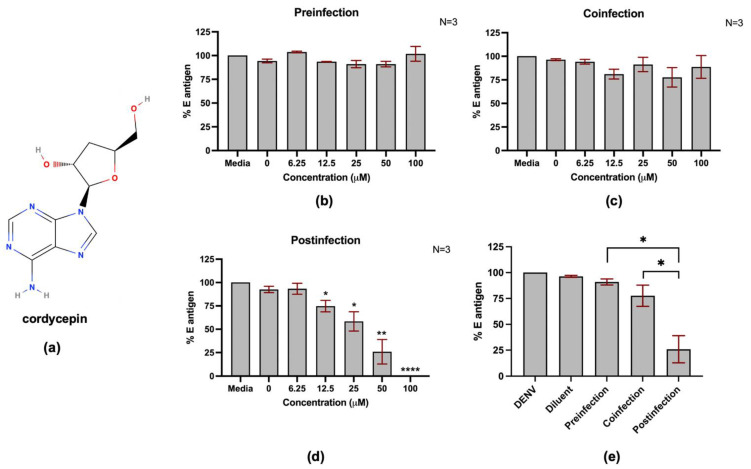
Anti-DENV activity of cordycepin in DENV2-infected Vero cells. Chemical structure of cordycepin (**a**). The inhibitory effect of cordycepin and its modes of action were determined by time-of-addition assay. (**b**–**d**) Vero cells were treated with cordycepin before (preinfection), during (coinfection), and after (postinfection) DENV2 infection. DENV2 envelope (E) protein levels were examined 48 h postinfection, and the percentages of DENV2 E protein levels relative to that of non-treatment control (set as 100%) were calculated. (**e**) The inhibitory effects of cordycepin at 50 μM were compared for different addition steps. (* indicates *p* < 0.05; ** indicates *p* < 0.01; and, **** indicates *p* < 0.0001.)

To test the hypothesis that cordycepin is able to inhibit DENV infection, Vero cells were infected with DENV2 and a time-of-addition assay was performed. The DENV2 envelope (E) protein in infected Vero cells was examined after the cells were treated with cordycepin at non-toxic concentrations (Figure S1) in different conditions. The Vero cells were treated with cordycepin (Figure 1a) at different times, including before, during, and after infection with DENV2, which were referred to as preinfection, coinfection, and postinfection, respectively. The results showed that treatment with cordycepin significantly lowered the DENV2 E protein level in only the postinfection condition. In the preinfection and coinfection conditions, cordycepin at the highest concentration tested (100 μM) did not change the DENV2 E protein level (Figure 1b–c). Additionally, treatment with cordycepin in the postinfection condition lowered DENV2 E protein levels in a dose-dependent manner (Figure 1d) at a half-maximal effective concentration (EC50) of 26.94 μM, as estimated by non-linear regression (Figure S2). The effects of cordycepin at 50 μM in different conditions were compared (Figure 1e). The results showed significant differences between postinfection treatment and the other conditions (Figure 1e). This result suggests that cordycepin inhibits DENV2 infection after cellular entry.

### 2.2. Cordycepin Reduced DENV2 Infection and Production

**Figure 2:**
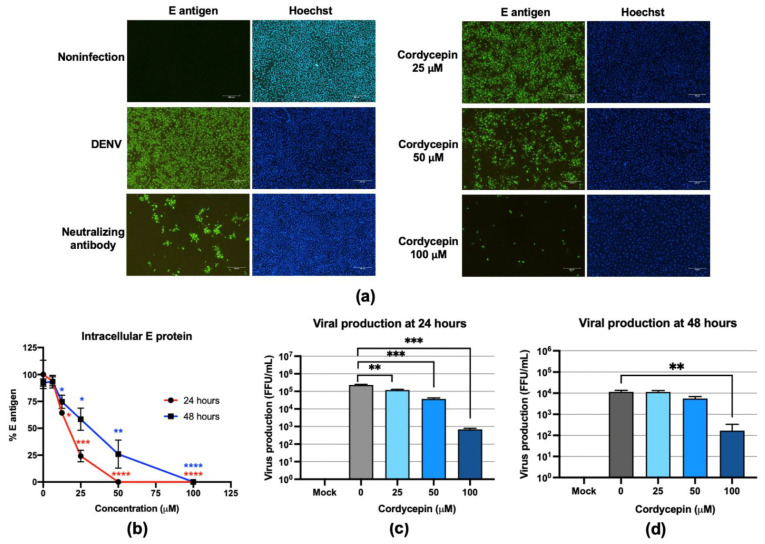
The inhibitory effects of cordycepin on DENV2-infected Vero cells, envelope (E) protein levels, and virus production in cell culture supernatants. (**a**) The numbers of DENV2-infected cells after treatment with cordycepin at concentrations of 25, 50, and 100 μM were examined 48 h postinfection by immunofluorescence assay (IFA). The DENV2 E protein was stained in green, and the nucleus was stained in blue. (**b**) The intracellular DENV2 E protein was examined by cell-based enzyme-linked immunosorbent assay (ELISA) 24 and 48 h after infection and cordycepin treatment. (**c**,**d**) DENV2 production in cell culture supernatants 24 and 48 h after infection and cordycepin treatment was determined by focus-forming assay. (* indicates *p* < 0.05; ** indicates *p* < 0.01; *** indicates *p* < 0.001, and **** indicates *p* < 0.0001.)

To further study the anti-DENV effect of cordycepin, we examined the number of DENV-infected cells, intracellular DENV E protein, and virus production in cell culture supernatants. Vero cells were infected with DENV2 and treated with cordycepin at concentrations of 25, 50, and 100 μM. The numbers of DENV-infected cells were then analyzed by immunofluorescence assay (IFA). The results showed a dose-dependent reduction in the numbers of DENV-infected cells 48 h after infection and cordycepin treatment (Figure 2a). The effects of cordycepin on intracellular DENV2 E protein levels 24 and 48 h after infection were compared. The results showed that cordycepin treatment at concentrations of 12.5–100 μM significantly reduced DENV2 E protein levels (Figure 2b), and the reduction was most pronounced 24 h after infection. DENV2 production in cell culture supernatants 24 and 48 h after infection was determined by focus-forming assay. The results demonstrated that DENV2 production was reduced both 24 and 48 h after infection and cordycepin treatment, but the reduction at 24 h was greater than that at 48 h (Figure 2c,d); 24 h after infection and cordycepin treatment, cordycepin at concentrations of 50 and 100 μM reduced the viral titers from 2.3 × 10^5^ to 3.4 × 10^4^ and 6.9 × 10^2^ FFU/mL, which represent approximately 6-fold and 300-fold reduction, respectively (Figure 2c).

### 2.3. Cordycepin Inhibits DENV2 RNA Replication and Molecular Docking of DENV2 NS5 Protein

**Figure 3:**
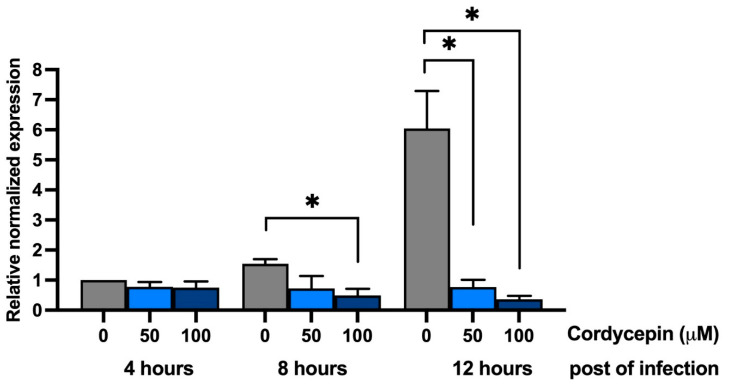
Effect of cordycepin on DENV2 RNA synthesis. Vero cells were infected with DENV2 and treated with cordycepin at concentrations of 0, 50, and 100 μM 4, 8, and 12 h postinfection. DENV2 RNA was quantitated by real-time reverse transcription polymerase chain reaction (RT-PCR). (* indicates *p* < 0.05.)

Since cordycepin (3′-deoxyadenosine) is an adenosine derivative which inhibits DENV infection at the early stage after its entry, we hypothesized that cordycepin also inhibits DENV infection at the step of RNA synthesis. To test this hypothesis, the effect of cordycepin on RNA synthesis was examined. DENV2-infected Vero cells were treated with cordycepin at concentrations of 0, 50, and 100 μM 4, 8, and 12 h after infection, and then the viral RNA was measured by real-time RT-PCR. In the condition without cordycepin treatment (0 μM), DENV2 RNA was increased in a time-dependent manner, and 12 h after infection, the relative amount of RNA was approximately 6-fold higher than the relative amount at 4 h (Figure 3). In contrast, in both cordycepin treatment conditions (50 and 100 μM), DENV2 RNA was slightly decreased 4 and 8 h after infection, and it was significantly decreased 12 h after infection. These results demonstrate that cordycepin at concentrations of 50 and 100 μM reduced RNA from approximately 6-fold to 0.77-fold and 0.36-fold, respectively.

**Figure 4:**
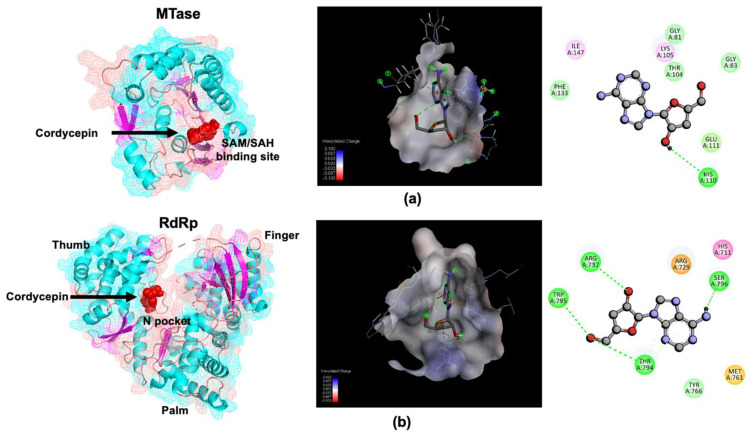
Molecular docking demonstrating cordycepin interaction with dengue NS5 protein. (**a**) The interaction of cordycepin with SAM/SAH binding site in the MTase domain was predicted (left and middle). Intermolecular interaction of cordycepin with the amino acid residues in the SAM/SAH binding site was shown in ball and stick (right) (**b**) The interaction of cordycepin with N pocket of DENV2 NS5 RNA-dependent RNA polymerase (RdRp) was predicted (left and middle). Intermolecular interaction of cordycepin with the amino acid residues in the N pocket was shown in ball and stick (right).

We also hypothesized that cordycepin can bind to DENV non-structural protein 5 (NS5), which is a crucial enzyme functioning in viral RNA synthesis. Thus, we initially performed molecular docking to simulate the interaction between cordycepin and NS5. Cordycepin was docked to the SAM/SAH binding site on the DENV2 NS5 MTase domain as the molecule was considered to have a similar structure to SAH. Comparable to the SAM/SAH binding site, the amino acids Gly81, Gly83, Thr104, Lys105, Phe133, and Ile147 were found in the binding pocket, as shown in Figure 4a. The complex structure showed high accuracy with more than 89% coverage of the key amino acids in the binding site and with a high binding affinity of −8.3 kcal/mol. The reported N pocket on the DENV2 NS5 RNA-dependent RNA polymerase (RdRp) domain was also targeted as a receptor of cordycepin according to its potential for small compound inhibitor binding. To validate the docking simulations, the co-crystalized ligand from the protein crystal was docked back to the binding pocket, as shown in the Figure S3. Cordycepin could bind to the site with a similar intermolecular hydrogen bonding to Arg737, Thr794, and TRP795, as shown in Figure 4b. In this case, the binding affinity of −5.9 kcal/mol was comparatively low, which might be due to its relatively small size. In addition, similar hydrophobic interactions could be obtained with Arg729, Tyr766, and Ser796.

### 2.4. Cordycepin Inhibits Infections of Four DENV Serotypes

**Figure 5:**
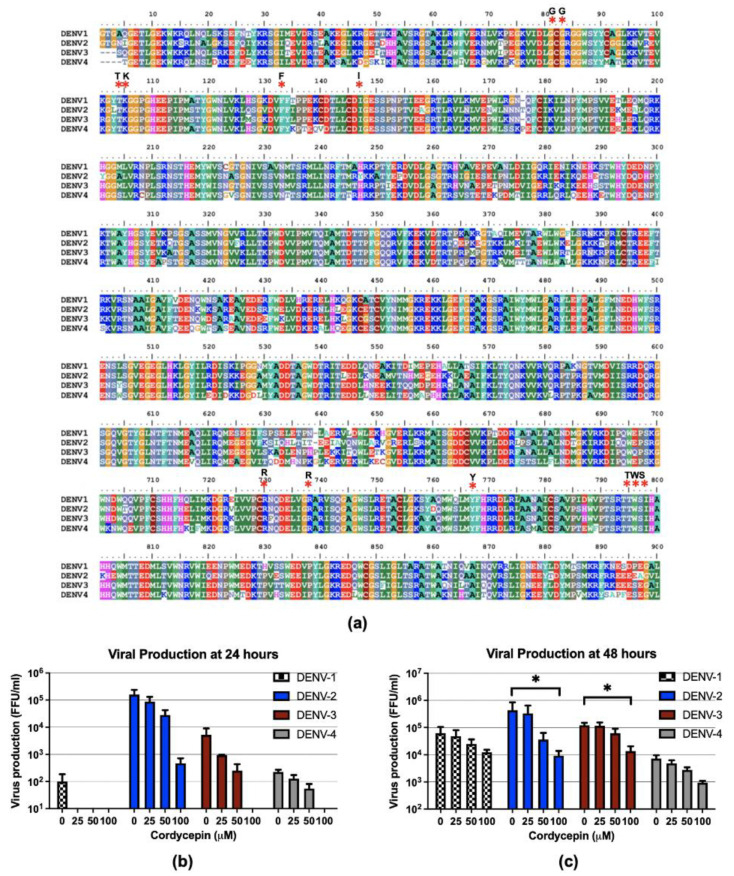
Anti-DENV activities of cordycepin on the inhibition of the four DENV serotypes. (**a**) Alignment of the amino acid sequences of the DENV NS5 protein from the four DENV serotypes was performed using the ClustalW multiple sequence alignment program of BioEdit software (Informer Technologies, Inc). The predicted cordycepin-binding amino acids across all four DENV serotypes are indicated by asterisks. (**b**,**c**) Virus production in Vero cells infected with DENV1, DENV2, DENV3, or DENV4 and treated with cordycepin at concentrations of 0, 25, 50, and 100 μM for 24 h (**b**) and 48 h (**c**) was determined by focus-forming assay. (* indicates *p* < 0.05.)

All four serotypes of DENV usually circulate in endemic areas, and secondary DENV infection with a serotype different from that which caused the primary DENV infection is associated with increased disease severity. Therefore, an anti-DENV agent that has cross-reactive effect among all DENV serotypes is required. To determine whether cordycepin exerts anti-DENV activity in different DENV serotypes, we first aligned the amino-acid sequences of DENV NS5, which is the hypothetical target of cordycepin, from all four DENV serotypes, including DENV1 (strain Hawaii), DENV2 (strain 16681), DENV3 (strain H87), and DENV4 (strain H241). This amino-acid sequence alignment showed 73.88% similarity (Figure 5a). Interestingly, the predicted amino-acid residues that bind to cordycepin, including Gly81, Gly83, Thr104, Lys105, Phe133, Ile147, Arg729, Arg737, Tyr766, Thr794, TRP795, and Ser796, were conserved among all four DENV serotypes (Figure 5a; indicated by asterisks). This finding highlights the possibility that cordycepin can bind to and inhibit all four DENV serotypes. We then examined the effects of cordycepin treatment on DENV production in Vero cells infected with each of the four DENV serotypes. The virus in the culture supernatant was determined by focus-forming assay after DENV infection for 24 and 48 h and cordycepin treatment at concentrations of 25, 50, and 100 μM. Reduced virus production was observed in a dose-dependent manner when the cells were infected with different DENV serotypes (Figure 5b,c). Significant reductions in virus production were observed when the cells were infected with DENV2 or DENV3 and treated with different doses of cordycepin. In contrast, non-statistically significant decreases in virus production were observed when the cells were infected with DENV1 or DENV4 and treated with different doses of cordycepin.

### 2.5. Cordyceps militaris Extract Inhibits DENV2 Infection

**Figure 6:**
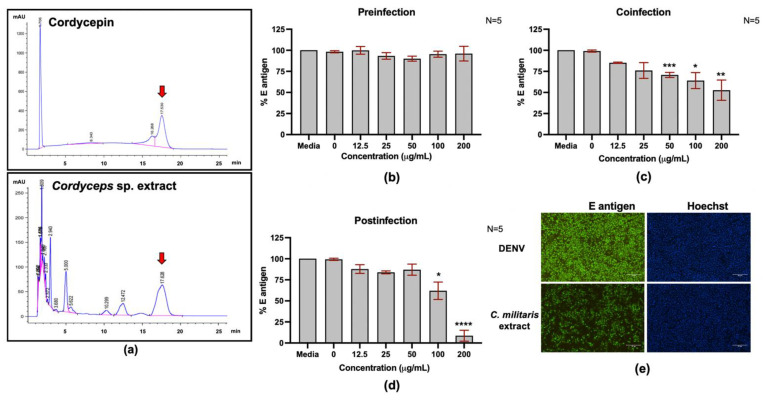
High-performance liquid chromatography (HPLC) profiles of cordycepin and *Cordyceps militaris* extract, and the anti-DENV2 activity of *C. militaris* extract in DENV2-infected Vero cells. (**a**) HPLC profiles of cordycepin (upper panel) and *C. militaris* extract (lower panel). (**b**–**d**) Anti-DENV2 activity of *C. militaris* extract in DENV2-infected Vero cells (**b**) before (preinfection), (**c**) during (coinfection), and (**d**) after (postinfection) DENV2 infection. (**e**) The DENV2 envelope (E) protein was detected 48 h postinfection by immunofluorescence assay (IFA). (* indicates *p* < 0.05; ** indicates *p* < 0.01; *** indicates *p* < 0.001, and **** indicates *p* < 0.0001.)

Cordycepin is a major bioactive compound present in *Cordyceps* spp., which has been used in Chinese medicine for centuries. *Cordyceps militaris* has also been cultivated in Thailand and other Asian countries. We questioned whether the *C. militaris* extract prepared in our laboratory would be able to inhibit DENV2 infection or not. To answer that question, a water extract of *C. militaris* was evaluated for its chemical ingredients by high-pressure liquid chromatography (HPLC). The results of that analysis revealed cordycepin to be a major chemical component, although other minor components also exist (Figure 6a). The cytotoxicity of *C. militaris* extract to the Vero cells was further examined. The cell viability after treatment with 50–800 μg/mL was determined and its sublethal dose at 200 μg/mL (cell viability >80%) was tested for anti-DENV2 activity (Figure S4). Time-of-addition assay was conducted to test the inhibitory effect of *C. militaris* extract in the preinfection (Figure 6b), coinfection (Figure 6c), and postinfection conditions (Figure 6d). Treatment of DENV2-infected cells with *C. militaris* extract at concentrations of 100 and 200 μg/mL could significantly reduce the DENV2 E protein in the coinfection and postinfection conditions (Figure 6c,d). The lowest reduction of E protein was observed in the postinfection treatment condition with cordycepin at 200 μg/mL, which could reduce the E protein level to 8.66% (Figure 6d).

## 3. Discussion

DENV infection is a major public health problem in many countries worldwide. Its prevalence has also rapidly increased during the past two decades with an approximately 8-fold increase from 505,430 in 2000 to 4.2 million in 2019, as reported by the World Health Organization (WHO) [1]. A vaccine is needed to prevent and control DENV infection in large populations; however, specific anti-DENV drugs are also needed to interrupt the infection cycle during outbreaks, to reduce disease severity, and to prevent mortality. Thus, the development of these vaccines and anti-DENV drugs is urgently required. To that end, our research group is actively working to identify anti-DENV agents that can be developed and applied for clinical use. Since cordycepin was reported to contain antiviral properties [13,14,15,16,17], we hypothesized that cordycepin and *Cordyceps* spp. extract possess anti-DENV activity. In this study, we found that cordycepin and *C. militaris* extract were able to inhibit DENV infection in DENV-infected Vero cells, and these findings suggest these substances as possible anti-DENV agents.

*Cordyceps* spp. is primarily and mostly an entomopathogenic fungus, which has been used in traditional Chinese medicine and is well known for its nutraceutical and therapeutic properties [18]. Cordycepin, which is a major bioactive compound isolated from *Cordyceps* spp., has gained interest for its pharmaceutical uses. The pharmaceutical potential of cordycepin has been reported in several disease models. The reported actions of cordycepin include antioxidant, anti-hyperlipidemia, anti-aging, anti-diabetes, anti-cancer, anti-malarial, antifungal, and antiviral. These reported findings emphasize the potential value of cordycepin and *Cordyceps* spp. extract for therapeutic applications. Cordycepin (3′-deoxyadenosine) is an adenosine derivative that lacks only a hydroxyl group at the 3′ position of the ribose moiety. Based on its basic structure, cordycepin likely binds to DNA and RNA polymerases, which are crucially required for viral replication. Cordycepin may also be incorporated into DNA and RNA strands to prematurely terminate their synthesis. We, therefore, hypothesized that cordycepin and *Cordyceps* sp. extract are able to inhibit DENV infection and production via inhibition of DENV RNA replication. The results of this study support our hypothesis. Cordycepin could significantly reduce the intracellular DENV envelope (E) protein in a dose-dependent manner, reflecting its ability to inhibit the DENV life cycle (Figure 1). The life cycle of DENV consists of sequential events beginning with DENV–host receptor binding, internalization, virus disassembly, protein synthesis, RNA synthesis, virus assembly, virus maturation, and ending with virus egress. Inhibition at a single step during the DENV life cycle may have an inhibitory effect on virus production. To single out the step of cordycepin action, we examined the effect of cordycepin in different sets of conditions, including preinfection, coinfection, and postinfection. The preinfection and coinfection conditions reflect the actions of cordycepin on DENV entry at the virus–host binding step and internalization, respectively, whereas the postinfection condition reflects virus disassembly and protein synthesis, and RNA replication (including the later steps of virus assembly and so on). The results of our experiments demonstrated obvious effects of cordycepin at the postinfection step (Figure 1), which suggests that cordycepin likely acted on RNA synthesis. The effect of cordycepin at the early stage of DENV infection resulted in a reduction of intracellular E protein levels and virus production in cell culture supernatants (Figure 2). Notably, the effect of cordycepin was absent in either preinfection or coinfection condition due to the removal of cordycepin from the system before and after the internalization step, respectively. Accordingly, this short incubation period might not be sufficient to allow the absorption of cordycepin into the cells to inhibit the RNA replication.

Based on our hypothesis that cordycepin binds to DENV RNA-dependent RNA polymerase and inhibits viral RNA synthesis, we further determined the levels of viral RNA in infected Vero cells after cordycepin treatment for 4, 8, and 12 h by the real-time RT-PCR method. In these conditions without cordycepin, the amounts of DENV2 RNA slightly changed 4 and 8 h after infection, but it significantly increased at 12 h (Figure 3). Interestingly, the treatment of infected cells with cordycepin at concentrations of 50 and 100 μM could clearly block DENV2 RNA synthesis. Since cordycepin is an adenosine derivative, it is likely that it can bind to the DENV NS5 protein (which is a key enzyme in viral RNA synthesis), which comprises 900 amino acids and has the following two functional domains: a methyltransferase (MTase) and an RNA-dependent RNA polymerase (RdRp) at its N-terminus and C-terminus, respectively. The NS5 MTase domain (residues 1–263) functions in capping genomic RNA using S-adenosylmethionine (SAM) as a methyl donor, and it is sequentially methylated at the N-7 position of guanine (m7G) and 2′ OH of ribose of the first strictly conserved adenosine nucleotide (Am) of RNA [19]. Another NS5 RdRp domain (residues 270–900) is responsible for de novo RNA synthesis without a primer requirement [19]. To initially examine the possibility that cordycepin can bind to NS5 and inhibit its action, we predicted the interaction of cordycepin and pocket sites on MTase and RdRp, which have been reported as antiviral agent target sites. The molecular docking results revealed interaction between cordycepin and MTase at the pocket that is naturally occupied by *S*-adenosyl-l-homocysteine (SAH) [20], with Δ*G* binding of −6.1 kcal/mol (Figure 4a). The molecular interaction revealed that cordycepin interacted with Gly81, His110, and Glu111 via intermolecular hydrogen bonding, and that competitive binding of cordycepin might disturb the natural ligand to the SAM/SAH binding site. During the process of methylation, MTase converts SAM as the methyl donor into SAH [21,22], and this process is an important replication activity. The SAM/SAH binding site on MTase has been proposed as a potential target for anti-flavivirus agents in both DENV [23] and Zika virus (ZIKV) [24]. Thus, the binding of cordycepin to the SAM/SAH binding site on MTase may inhibit DENV RNA replication.

Furthermore, the ability of cordycepin to bind to the RdRp domain was also predicted by molecular docking. The result showed that cordycepin could bind to Arg737, Thr794, and TRP795 via hydrogen bonding, and to Arg729, Tyr766, and Ser796 via hydrophobic interactions with Δ*G* binding of −5.9 kcal/mol (Figure 4b). This binding pocket, namely N pocket, was discovered by fragment-based screening via X-ray crystallography targeting the apo-RdRp [25,26]. The reported active compound, namely compound 29, which specifically interacted with this pocket, was previously shown to efficiently inhibit RdRp activity, and to reduce DENV replication in BHK21, Huh7, and A549 cells [27]. Although the Δ*G* binding values of cordycepin to these two candidate sites were lower than those of the natural ligands, cordycepin probably bound to multi-target sites that might confer additive or synergistic effects that contribute to its inhibitory activity. Interestingly, the function of MTase was reported to enhance both stability and activity at the initiation and elongation steps of the RdRp domain [28,29]. Inhibition of these two domains that function so interdependently may strongly influence viral RNA replication. The antivirus effect of cordycepin against Epstein-Barr virus (EBV) [13,14], influenza viruses [15,16], and human immunodeficiency virus (HIV) [17] has been reported. The mechanism of cordycepin was demonstrated to inhibit reverse transcriptase of HIV [17], which was the direct effect on the viral protein. However, in Epstein–Barr virus, the action of cordycepin was to modulate signal transduction and epigenetic modification, which are related to the regulation of virus infection [13], highlighting the indirect effect of cordycepin via host protein modulation. Notably, cordycepin has been studied and is known to modulate a variety of cellular signaling pathways involved in apoptosis, proliferation, angiogenesis, and inflammation [30]. Thus, the indirect effects of cordycepin on the host proteins involved in host antiviral response cannot be excluded. Further in vitro or in vivo studies should focus on both the direct effects of cordycepin on viral proteins, and the indirect effects of cordycepin on host proteins in the inhibition of DENV infection and replication.

The amino acid sequences of the NS5 proteins from the four DENV serotypes are highly conserved. Accordingly, the cordycepin-interacting amino-acid residues on the MTase and RdRp domains are also highly conserved among the four DENV serotypes (Figure 5a). We, therefore, examined the anti-DENV activities of cordycepin against all four DENV serotypes. The observed cordycepin-induced inhibition of virus production was shown to be dose-dependent in all four DENV serotypes. The highest inhibition of virus production was observed in DENV2, followed by DENV3 (both *p* < 0.05). In contrast, the decreases in virus production were non-statistically significant in DENV1 and DENV4 (Figure 5b). The observed differences in antiviral activity among the four DENV serotypes might be explained by variations in the amino acids lining the binding pocket. Although the key binding amino acid residues were conserved, variation in the other amino acids might affect the accessibility or stabilization of cordycepin inside the pocket. To improve binding affinity, in silico prediction of an additional chemical moiety could be applied, which is based on the size of cordycepin and the area occupied in the pocket. Other derivatives or secondary metabolites of cordycepin might also be selected as alternative molecules for a binding affinity improvement strategy.

*Cordyceps* spp. extract has been used in traditional medicine and nutraceuticals in Thailand and other Asian countries. Since cordycepin is known to be the major bioactive compound in *Cordyceps* spp., we also conducted experiments to evaluate the effects of *C. militaris* extract on anti-DENV activity. The amount of cordycepin in *C. militaris* extract was examined by HPLC, and the area under the curve (AUC) was calculated. Cordycepin was present at approximately 10.72 mg/g of *C. militaris* extract. Treatment with *C. militaris* extract at 200 μg/mL (containing about 2 ng/mL or 7.96 μM cordycepin) significantly reduced the intracellular DENV2 E protein in the postinfection condition, which is similar to the reduction observed after cordycepin treatment. Unexpectedly, a reduction in DENV2 E protein was also observed in the coinfection condition, and this finding was not observed after treatment with cordycepin. This suggests an additional effect of *C. militaris* extract, which is different from the pure cordycepin compound. Since *C. militaris* extract contains a combination of many compounds, the observed inhibitory activity in the coinfection condition might be due to the collective effects of this mixture of compounds. Hence, it is possible that the anti-DENV activity of *C. militaris* extract was caused by the combination of at least two or more compounds or agents that inhibited both virus entry and replication. Since DENV infection and its effects on host cells are highly complex and dynamic, the inhibition of DENV infection via different modes of action by targeting multiple steps in the DENV life cycle is a compelling strategy for developing anti-DENV drugs. For example, our group recently reported peptide inhibitors targeting DENV entry by inhibiting virus–host receptor interaction [31,32,33,34]. A combination of these peptide inhibitors and cordycepin would hypothetically enhance antiviral activity in DENV-infected cells. Further studies should be conducted to test this hypothesis.

Natural compounds from medicinal plants are excellent sources for screening anti-DENV and other antiviral drugs. Additionally, the processes of drug discovery and development can be shortened and accelerated, respectively, due to the already known therapeutic properties and proof-of-safety based on long-standing traditional use. However, the modes of action and molecular mechanisms of the bioactive compounds identified in and isolated from medicinal plants need to be thoroughly investigated before they can be assigned to specific clinical applications.

In conclusion, our study demonstrated the anti-DENV activity of cordycepin and *C. militaris* extract. Their inhibitory effects take place at the early stage of DENV infection, and they are involved in the step of viral RNA synthesis. The binding of cordycepin to the MTase and RdRp domains of DENV NS5 has been proposed, and is illustrated by molecular docking. The therapeutic potential of cordycepin for DENV infection should be further investigated for future clinical application as an anti-DENV drug.

## 4. Materials and Methods

### 4.1. Cell Culture and Virus Propagation

Monkey kidney epithelial Vero cells (CCL8; American Type Culture Collection (ATCC), Manassas, VA, USA) were cultured in minimal essential medium (MEM) supplemented with 10% (*v*/*v*) fetal bovine serum (FBS) (Gibco; Thermo Fisher Scientific, Waltham, MA, USA) and penicillin/streptomycin (Gibco, Thermo Fisher Scientific, Waltham, MA, USA). The cells were incubated at 37 °C in a 5% CO_2_ atmosphere. Four serotypes of DENV, including DENV1 (strain Hawaii originally from Hawaii, USA), DENV2 (strain 16,681 originally from Thailand), DENV3 (strain H87 originally from Thailand), and DENV4 (strain H241 originally from the Philippines), were propagated in C6/36 cells. Cell supernatants containing DENV were collected, and the virus titers were quantitated by focus-forming assay. The virus was stored at −70 °C until use.

### 4.2. Time of Addition Assay

Cordycepin or *C. militaris* extract was tested for its mode of action during the virus life cycle using time-of-addition assay. Briefly, Vero cells were plated in 96-well format (2 × 10^4^ cells per well) overnight before the experiment. At the time of experiment, cordycepin or *C. militaris* extract at the indicated concentrations was added to the cells in three different conditions, including preinfection, coinfection, and postinfection (Figure S5). To test the possibility that cordycepin or *C. militaris* extract affected on the host cell receptor binding step or the virus entry step, the preinfection or coinfection condition was accordingly performed. The preinfection condition was carried out by adding cordycepin or *C. militaris* extract for 30 min and by taking the culture medium out and washing before infection with 1.0 × 10^4^ FFU/mL of DENV2 (100 μL), whereas in the coinfection condition, cordycepin or *C. militaris* extract and DENV were simultaneously added. After incubation for 2 h to allow the virus binding and internalization, the unbound viruses and cordycepin in the extract were removed. Then, the infected cells were washed once with cold PBS and replaced with a fresh medium. The postinfection condition was performed after the cell–virus incubation for 2 h, addition of cordycepin or *C. militaris* extract and its maintenance along the experiment to test the effect of cordycepin or *C. militaris* extract on protein or RNA synthesis (including the later steps of virus assembly and so on) after the virus entry step. The infected cells were harvested 48 h after infection to determine the level of intracellular DENV E protein by cell-based enzyme-linked immunosorbent assay (ELISA).

### 4.3. Virus Titration and Focus Forming Unit (FFU) Staining

To examine the anti-DENV activity of cordycepin or *C. militaris* extract on virus production, the newly synthesized virus in the culture supernatant was measured by FFU staining. Vero cells were plated in 96-well format (2 × 10^4^ cells per well) overnight before the experiment. The cells were infected with DENV1, DENV2, DENV3, or DENV4 (1.0 × 10^4^ FFU/mL of DENV2, 100 μL). The plate was incubated for 2 h to allow the virus to enter the cells. The unbound virus was removed and a fresh medium containing 25, 50, or 100 μM of cordycepin was added to the cells. The new viral progenies were harvested at 24 or 48 h, and determined for their virus titer by FFU staining.

To determine DENV titers, the Vero cells were plated 2 × 10^4^ cells per well in 96-well format. A 10-fold serial dilution of DENV was added to the cells. After 2 h, the infected cells were overlaid with 2% carboxymethylcellulose medium, and the plate was further incubated for 72 h. The infected cells were fixed and permeabilized using 3.8% formaldehyde and 0.2% Triton X-100, respectively. The 4G2 monoclonal antibody, which is specific to DENV E proteins, was added to the cells and the plate was incubated overnight. The cells were washed three times with phosphate-buffered saline (PBS) containing 0.1% Tween-20 (PBST) before addition of the horseradish peroxidase (HRP)-conjugated secondary antibody (at 1:2000 dilution), which was incubated at room temperature (RT) for 30 min. The DENV-infected cells shown as DENV foci were detected by adding 3,3′-diaminobenzidine (DAB) substrate (Sigma-Aldrich Corporation, St. Louis, MO, USA). The DENV foci were counted manually under a light microscope (20 × magnification) (Nikon Instruments, Inc., Melville, NY, USA).

### 4.4. Cell-Based Enzyme-Linked Immunosorbent Assay (ELISA)

Infected cells from the time-of-addition assay were examined for their level of intracellular DENV E protein. The infected cells were collected at the indicated time and washed once with PBS. The cells were fixed and permeabilized using 3.8% formaldehyde and 0.1% Triton X-100, respectively, before addition of the 4G2 monoclonal antibody specific to the DENV E protein. The plate was incubated overnight and washed three times with PBST. HRP-conjugated secondary antibody (dilution 1:2000) was then added to the wells and incubated at RT for 30 min. The plate was washed three times with PBST before adding the 3,3′,5,5′-tetramethylbenzidine substrate (Invitrogen Corporation, Carlsbad, CA, USA). Absorbance was detected and calculated for the percentage of DENV E protein. The absorbance percentage for infected cells without treatment (control) was set as 100%.

### 4.5. Immunofluorescence Assay (IFA)

The number of DENV-infected cells was detected by IFA. Briefly, the cells were plated in 24-well format (1.0 × 10^5^ cells per well) overnight before the experiment. On the day of the experiment, the cells were infected with 1.0 × 10^4^ FFU/mL of DENV2 (500 μL). The plate was incubated with the viruses for 2 h to allow virus entry into the cells. The unbound viruses were removed and replaced with a fresh medium containing cordycepin at concentrations of 25, 50, and 100 μM; 48 h after infection, the infected cells were harvested before being fixed and permeabilized using 3.8% formaldehyde and 0.1% Triton X-100, respectively. After that, a 4G2 monoclonal antibody specific to DENV E protein was added. The cells were washed three times with PBST, and Alexa Fluor 488 goat anti-mouse IgG (Invitrogen) was added in combination with Hoechst^®^ 33,342 nucleic acid stain (Invitrogen). DENV-infected cells were monitored under a fluorescence microscope.

### 4.6. Real-Time Polymerase Chain Reaction (RT-PCR)

To investigate the effect of cordycepin on DENV replication, Vero cells were seeded at 1.5 × 105 cells per well in a 12-well plate for 24 h before addition of DENV2 for 2 h at 37 °C with 5% CO_2_. The unbound viruses were removed and cordycepin at concentrations of 50 and 100 µM was added. The cells were harvested at 4, 8, and 12 h, and the total RNA was extracted using TRIzol™ Reagent (Invitrogen). The RNA (1.0 µg) was converted into cDNA using a cDNA Synthesis Kit (Toyobo Life Science, Osaka, Japan). The PCR mixture contained 0.5 µg of cDNA, DENV E-specific primers [10 µM of D2R (5′-CCGGCTCTACTCCTATGATG-3′), and 10 µM of D2L (5′-ATCCAGATGTCATCAGGAAAC-3′)], and 2 × SensiFAST SYBR No-ROX Mix (Meridian Bioscience, Cincinnati, OH, USA). RT-PCR was then performed on an iCycler Thermal Cycler (Bio-Rad Laboratories, Hercules, CA, USA). The data were normalized with the housekeeping gene GAPDH and analyzed for relative value to that of non-treated control (set as 1).

### 4.7. Molecular Docking

Molecular docking aimed to predict the interaction between cordycepin and the structures of DENV NS5 MTase and RdRp. In this study, the three-dimensional structure of cordycepin was generated from its crystal structure (CCDC number 129360) in MOL2 format. Hydrogens were added, non-polar hydrogens were merged, and the missing atoms were added and converted from MOL2 to pdbqt format using AutoDock Tools version 1.5.6 [35].

The crystallographic coordinates of DENV NS5 MTase and RdRp were retrieved from the Protein Data Bank (PDB ID: 1R6A, 5I3P, 5I3Q, and 5K5M). The water molecules, ions, or inhibitors in each structure were removed to prepare a receptor target model in docking simulations. Grid box sizes of 30 × 40 × 40 and 20 × 20 × 24 Å at a spacing of 1 Å were generated by defining the ligand binding site (SAM binding site, co-crystalized ligand or equivalent positions in the different protein sequences as centroid). The docking procedure was performed using Autodock Vina with rigid docking. A complex structure with favorable binding affinity was selected, and molecular interactions of the ligand and the protein were analyzed using LigPlot version 2.2.4 [36]. The docking poses were prepared using the PyMOL Molecular Graphics System, Version 2.0 (Schrödinger, LLC.; New York, NY, USA).

### 4.8. Cordyceps Extraction

Dried fruiting bodies that included substrates of *C. militaris* were obtained from AAVA Group Co., Ltd., Bangkok, Thailand. The sample was ground and soaked with sterile distilled water at a ratio of 1:10 (w/v). The sample was macerated with distilled water at 45 °C for 3 h. The extract was filtered using Whatman No. 1 filter paper and then evaporated using a rotary evaporator at 45 °C under a reduced pressure of 50 mbar. The extract was dried by lyophilization and stored at −20 °C until use.

### 4.9. High Performance Liquid Chromatography (HPLC)

Crude extract of *C. militaris* was analyzed by high-performance liquid chromatography (HPLC). A pure compound of cordycepin (Sigma-Aldrich) was purchased to optimize the detection of the cordycepin component in *C. militaris* extract using an isocratic HPLC system with a conventional C18 column. The extract was filtered through a 0.45 µm microfilter, and 50 µL of the filtrate was injected into the HPLC system (Agilent Technologies, Santa Clara, CA, USA). A ZORBAX Eclipse XDB-C18 column (4.6 × 150 mm, 5 µm; Agilent Technologies) employed an ultraviolet (UV) photodiode array detector (254 nm). The HPLC system was controlled to have a flow rate of 1.0 mL per minute, and a running time of 30 min at 31 °C. Cordycepin in *C. militaris* extract was separated by water and methanol in the ratio of 92:8 (*v/v*). The bioactive compound profiles of *C. militaris* extract were analyzed by comparing them with those of standard cordycepin.

### 4.10. Statistical Analysis

The datasets were analyzed, and the results are shown as the mean ± SEM of at least three independent experiments. The statistical analysis was performed using a Student’s *t*-test in GraphPad Prism software, version 9.0.2 (GraphPad Software, Inc., San Diego, CA, USA). The symbols *, **, ***, **** indicate *p*-values of <0.05, <0.01, <0.001, and <0.0001, respectively.

## Data Availability

Not available.

## Supplementary Materials

The following data are available: Figure S1: The cell cytotoxicity of cordycepin; Figure S2: The half maximal effective concentration (EC50) of cordycepin on the inhibition of DENV2 infection; Figure S3: Superpositions of residues in binding pocket of X-ray structure and simulated acyl-sulfonamide derivative bound to the N pocket of DENV2 NS5 RNA dependent RNA polymerase (RdRp); Figure S4: The cell cytotoxicity of *Cordyceps militaris* extract; Figure S5: The schematic diagram of time of addition assay.

## Sample Availability

Not available.
