# Supplementary material for: Cordycepin Inhibits Virus Replication in Dengue Virus-Infected Vero Cells
Source: Molecules. 2021 May 23;26(11):3118. doi: 10.3390/molecules26113118 (PMC8197141; doi:10.3390/molecules26113118)
Supplement: Supplementary file 1 [file molecules-26-03118-s001.zip › molecules-1220797-supplementary.pdf]

**Supplementary Materials:**

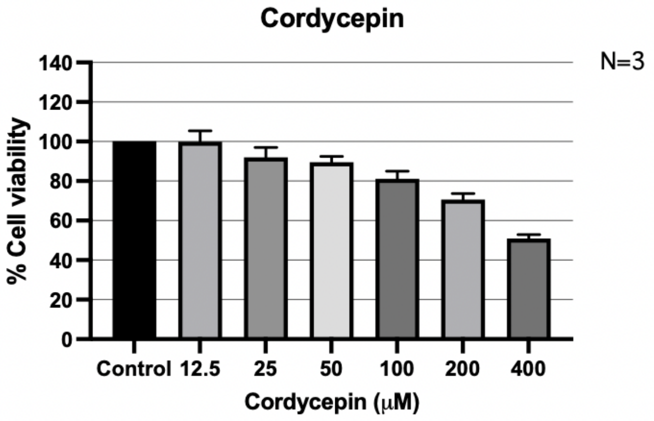


**Figure S1. The cell cytotoxicity of cordycepin.** The cell viability of Vero cells were measured after 48-hour treatment with cordycepin at various concentrations ranging from 3.125-400 μM by using PrestoBlue ™ reagent (Invitrogen, MA, USA). The data from three experiments were analysed and represented as the % cell viability relative to that of non-treatment control which set as 100%.

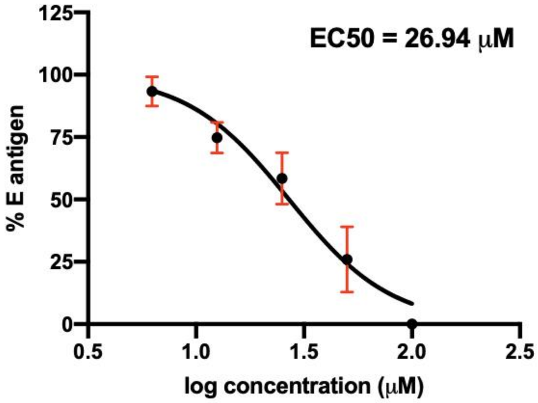


**Figure S2. The half maximal effective concentration (EC50) of cordycepin on inhibiting DENV2.** The Vero cells were infected with DENV2 and treated with various concentrations of cordycepin. At 48 hours after infection, the cells were harvested and examined the intracellular E antigen by using cell-based ELISA. The data from three experiments were calculated to % E antigen relative to that of non-treatment control (set as 100%). The % E antigen was analyzed for EC50 values by using non-linear regression of GraphPad Prism software version 9.0.2 (GraphPad Software, CA, USA).

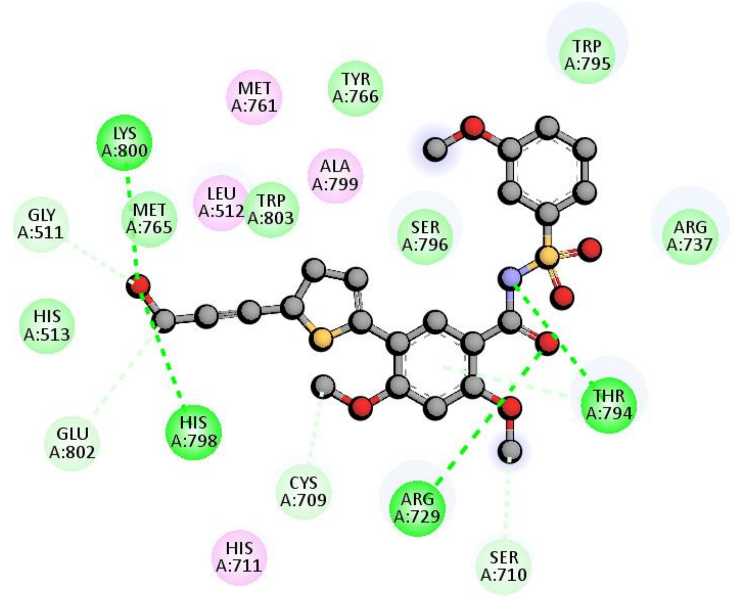


**Figure S3.** Superpositions of residues in binding pocket of X-ray structure and simulated acyl-sulfonamide derivative bound to the N pocket of DENV2 NS5 RNA dependent RNA polymerase (RdRp)

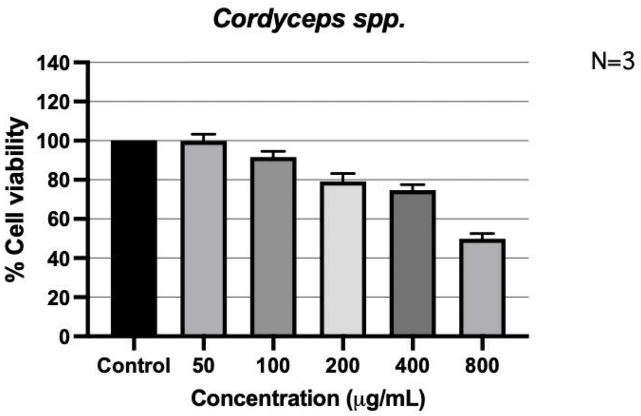


**Figure S4. The cell cytotoxicity of *Cordyceps militaris* extract.** The cell viability of Vero cells were measured after 48-hour treatment with *C. militaris* at various concentrations ranging from 3.125-400 μM by using PrestoBlue ™ reagent (Invitrogen, MA, USA). The data from three experiments were analysed and represented as the % cell viability relative to that of non-treatment control which set as 100%.

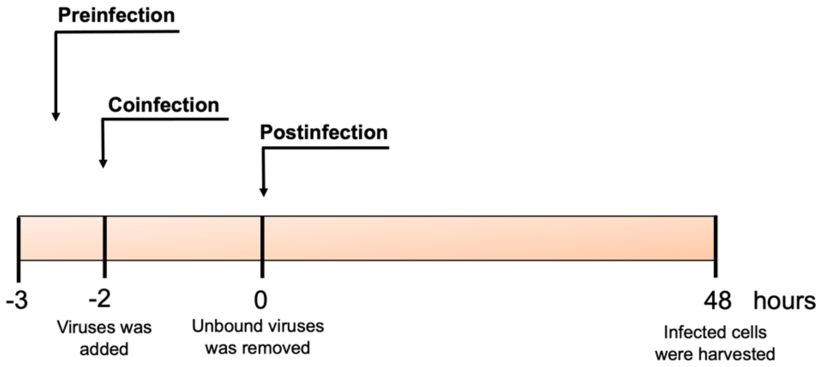


**Figure S5. The schematic diagram of time of addition assay.** The preinfection step was carried out by adding cordycepin or *C. militaris* extract for 30 minutes, removing, and washing out before infection with 1.0 x $10^4$ FFU/mL of DENV2 (100 μL) whereas in the coinfection step, cordycepin or *C. militaris* extract and DENV were simultaneously added. After incubation for 2 hours to allow the virus binding and internalization, the unbound viruses were removed, washed, and added with fresh medium. The postinfection step was performed by cell-virus incubation for 2 hours, addition of cordycepin or *C. militaris* extract and its maintenace along the experiment. The infected cells were harvesred at 48 hours after the infection.
